# Very early MRI responses to therapy as a predictor of later radiographic progression in early rheumatoid arthritis

**DOI:** 10.1186/s13075-019-2000-1

**Published:** 2019-10-21

**Authors:** Philip G. Conaghan, Mikkel Østergaard, Orrin Troum, Michael A. Bowes, Gwenael Guillard, Bethanie Wilkinson, Zhiyong Xie, John Andrews, Amy Stein, Douglass Chapman, Andrew Koenig

**Affiliations:** 1 Leeds Institute of Rheumatic and Musculoskeletal Medicine, University of Leeds, and UK National Institute for Health Research Leeds Biomedical Research Centre, Leeds, UK; 2 Copenhagen Center for Arthritis Research, Center for Rheumatology and Spine Diseases, Rigshospitalet, Glostrup, and Department of Clinical Medicine, University of Copenhagen, Copenhagen, Denmark; 3 Division of Rheumatology, University of Southern California Keck School of Medicine, Santa Monica, CA USA; 4 Imorphics Ltd, Worthington House, Towers Business Park, Manchester, UK; 5 Pfizer Inc, Groton, CT USA; 6 Pfizer Inc, New York, NY USA; 7 Biostatistics Department, IQVIA Inc, Morrisville, NC USA; 8 Pfizer Inc, Collegeville, PA USA

**Keywords:** Bone erosion, Disease activity, Joint space narrowing, Osteitis, Predictive ability, Radiographic progression, RAMRIS, RAMRIQ, Rheumatoid arthritis, Synovitis

## Abstract

**Background:**

The objective of this study was to evaluate early changes in magnetic resonance imaging (MRI) and clinical disease activity measures as predictors of later structural progression in early rheumatoid arthritis (RA).

**Methods:**

This was a post hoc analysis of data pooled across treatments from a three-arm (tofacitinib monotherapy, tofacitinib with methotrexate [MTX], or MTX monotherapy) trial of MTX-naïve patients with early, active RA. Synovitis, osteitis and erosions were assessed with the Outcome Measures in Rheumatology (OMERACT) RA MRI scoring system (RAMRIS) and RAMRIQ (automated quantitative RA MRI assessment system; automated RAMRIS) at months 0, 1, 3, 6 and 12. Radiographs were assessed at months 0, 6 and 12, and clinical endpoints were assessed at all timepoints. Univariate and multivariate analyses explored the predictive value of early changes in RAMRIS/RAMRIQ parameters and disease activity measures, with respect to subsequent radiographic progression.

**Results:**

Data from 109 patients with a mean RA duration of 0.7 years were included. In univariate analyses, changes in RAMRIS erosions at months 1 and 3 significantly predicted radiographic progression at month 12 (both *p* <  0.01); changes in RAMRIQ synovitis and osteitis at months 1 and 3 were significant predictors of RAMRIS erosions and radiographic progression at month 12 (all *p* <  0.01). In subsequent multivariate analyses, RAMRIS erosion change at month 1 (*p* <  0.05) and RAMRIQ osteitis changes at months 1 and 3 (both *p* <  0.01) were significant independent predictors of radiographic progression at month 12. Univariate analyses demonstrated that changes in Clinical Disease Activity Index (CDAI) and Disease Activity Score in 28 joints, erythrocyte sedimentation rate (DAS28-4[ESR]) at months 1 and 3 were not predictive of month 12 radiographic progression.

**Conclusions:**

MRI changes seen as early as 1 month after RA treatment initiation have the potential to better predict long-term radiographic progression than changes in disease activity measures.

**Trial registration:**

ClinicalTrials.gov, NCT01164579.

## Background

Rheumatoid arthritis (RA) has a substantial impact on patients, with joint inflammation resulting in pain and destruction of the joints, leading to reduced quality of life and substantial disability [1]. A treat-to-target approach is recommended in RA, with regular assessments and adjustment of treatment for inadequate response [2, 3]. Studies have demonstrated clinical response to therapy at months 1 to 3 to be predictive of longer-term achievement of clinical targets (decrease in Disease Activity Score in 28 joints, erythrocyte sedimentation rate [DAS28-4(ESR)] and Clinical Disease Activity Index [CDAI]) with conventional synthetic disease-modifying antirheumatic drugs (DMARDs) [4], biologic DMARDs [5] and the Janus kinase (JAK) inhibitor, baricitinib [6].

Magnetic resonance imaging (MRI) can assess inflammation (synovitis and bone marrow edema/osteitis) as well as damage (erosions) in joints, using the semiquantitative Outcome Measures in Rheumatology (OMERACT) RA MRI scoring system (RAMRIS) [7, 8] and, more recently, the automated quantitative RA MRI assessment system (RAMRIQ; automated RAMRIS) [8, 9]. This sensitivity for pathology detection may provide a tool for accurate early detection of an objective treatment response.

Tofacitinib is an oral JAK inhibitor for the treatment of RA. In a phase 2 study of tofacitinib 10 mg twice daily (BID) with and without concomitant methotrexate (MTX), tofacitinib reduced inflammation and inhibited progression of structural damage from baseline to month 12 compared with treatment with MTX alone, as assessed using RAMRIS and RAMRIQ [8].

The aim of this post hoc analysis was therefore to evaluate early changes in MRI and clinical measures in this phase 2 study as predictors of later structural changes.

## Methods

### Study design and patients

This was a post hoc analysis of data from a 1-year, exploratory, phase 2, randomised, double-blind, parallel-group study (A3921068; NCT01164579). Full details of the study design and patient population have been reported previously [8]. Briefly, patients aged ≥ 18 years with active RA were randomised 1:1:1 to tofacitinib 10 mg BID monotherapy, tofacitinib 10 mg BID with MTX, or MTX monotherapy. Patients had RA of ≤ 2 years’ duration and 103/109 (94%) patients were MTX-naïve. This study was approved by the Institutional Review Boards and/or Independent Ethics Committees at each investigational centre, and all patients provided written informed consent.

### MRI and radiography

MRIs of unilateral wrist and metacarpophalangeal (MCP) joints were obtained at screening and months 1, 3, 6 and 12, and were used to assess synovitis, osteitis (bone marrow oedema) and erosions using RAMRIS [7] and RAMRIQ [9]. The hand that was most clinically affected at screening was chosen for MRI assessment throughout the study. If both hands were equally affected, the dominant hand was chosen. Radiographic progression was based on mean change from baseline in modified Total Sharp Scores (mTSS) [10], determined from radiographs at baseline, month 6 and month 12. The OMERACT RAMRIS semiquantitatively assessed osteitis (using short-tau inversion recovery sequences), synovitis (using pre/post-contrast T1-weighted sequences) and bone erosions (using T1-weighted sequences) in three areas of the wrist and in MCP joints 1–5 [7], using a single, centralised reader, blinded to chronology and all clinical and imaging data. RAMRIQ is a fully automated quantitative measurement system (Imorphics, Manchester, UK) that assessed the same three pathologies as RAMRIS. For RAMRIQ assessments, the pre-contrast and post-contrast T1-weighted MRI sequences of the same joints and bones were used. The system is based on the accurate 3D segmentation of finger bones using a method based on statistical shape models that identify the whole bone in 3D and are therefore less susceptible to differences between readers and hand position in the MRI coil. Synovitis volume is the volume of the regions around the joint where the synovial capsule is found that enhances with contrast agent on T1-weighted images. Osteitis volume is the volume inside the bone that enhances with contrast agent on T1-weighted images. Finally, erosions are measured as the volume of bone (either the complete bone, or the bone within 15 mm of the joint [10 mm for RAMRIS] for longer bones) that is classified as ‘not healthy’ using a machine-vision classification method. RAMRIQ osteitis values were reported as normalised volume (%) calculated as (total volume osteitis/total bone volume) × 100, and RAMRIQ erosions values were also reported as normalised volume (%), calculated as (total volume eroded bone/total bone volume) × 100.

### Disease activity

Disease activity endpoints were changes from baseline at months 1 and 3 in CDAI scores and DAS28-4(ESR).

### Statistical methods and analysis

Analyses were on data pooled across treatment groups, given the sample size. Pooled analyses were performed, as reliable markers of RA progression should not differ in their predictive value across treatment groups; thus, pooled analyses allow for more generalisation.

Univariate linear regression analyses were used to assess the predictive value of changes in RAMRIS synovitis, osteitis and erosions from baseline to month 1 and, separately, from baseline to month 3, with respect to radiographic progression (mTSS) from baseline to month 12. Additionally, univariate linear regression analyses were used to assess the predictive value of changes in RAMRIQ synovitis, osteitis and erosions from baseline to month 1 and, separately, from baseline to month 3, with respect to changes in RAMRIS erosions and radiographic progression between baseline and month 12. Based on the results of the univariate analyses, we performed subsequent multivariate linear regression analyses of changes from baseline to month 1 and, separately, from baseline to month 3, with respect to radiographic progression from baseline to month 12, which included the RAMRIS and RAMRIQ variables that were shown to be statistically significant predictors in the univariate analyses. Univariate analyses were also used to assess the predictive value of changes in CDAI and DAS28-4(ESR) from baseline to month 1 and, separately, from baseline to month 3, with respect to radiographic progression from baseline to month 12. Statistical significance was considered to have been demonstrated with *p* values < 0.05; multiplicity was not adjusted for.

## Results

### Patients

Overall, 109 patients, 103 of whom were MTX-naïve, with mean ± standard deviation duration of RA of 0.7 ± 1.0 years were randomised, 36 to each tofacitinib 10 mg BID group (± MTX) and 37 to MTX monotherapy. Patient demographics and baseline disease characteristics have been reported previously and were generally well-balanced between treatment groups [8].

### Radiographic progression

mTSS at baseline, month 6 and month 12, and least squares mean changes from baseline in mTSS at month 6 and month 12, are reported in Additional file 1.

### Early RAMRIS changes as predictors of radiographic progression and joint space narrowing (JSN)

In univariate analyses, lower increases from baseline to both months 1 and 3 in RAMRIS erosions were significant predictors of less radiographic progression from baseline to month 12. Furthermore, lower increases from baseline to both months 1 and 3 in RAMRIS erosions significantly predicted less radiographic erosion from baseline to month 12, and lower increases from baseline to month 1 in RAMRIS erosions significantly predicted less JSN from baseline to month 12. No significant predictive value was observed between change from baseline to month 1 or 3 in RAMRIS synovitis or osteitis or between baseline and month 12 in radiographic progression, radiographic erosions, or JSN (Table 1).

**Table 1:** Univariate analyses^a^ of predictive value of early MRI changes for later MRI and radiographic changes

		Change from baseline to month 1			Change from baseline to month 3		
Change from baseline to month 12		RAMRIS synovitis	RAMRIS osteitis	RAMRIS erosions	RAMRIS synovitis	RAMRIS osteitis	RAMRIS erosions
Total radiographic progression^b^	Estimate (SE); *p* value	0.044 (0.241); 0.856	0.007 (0.175); 0.971	1.431 (0.408); < 0.001	0.101 (0.188); 0.592	− 0.087 (0.138); 0.533	0.748 (0.252); 0.004
Radiographic erosion progression	Estimate (SE); *p* value	0.039 (0.118); 0.743	0.031 (0.085); 0.718	0.638 (0.201); 0.002	0.087 (0.091); 0.339	− 0.013 (0.067); 0.853	0.455 (0.118); < 0.001
Radiographic JSN progression	Estimate (SE); *p* value	0.010 (0.163); 0.951	− 0.024 (0.118); 0.837	0.792 (0.284); 0.007	0.015 (0.127); 0.905	− 0.074 (0.093); 0.434	0.295 (0.179); 0.104
		RAMRIQ synovitis^c^	RAMRIQ osteitis	RAMRIQ erosions	RAMRIQ synovitis^c^	RAMRIQ osteitis	RAMRIQ erosions
RAMRIS erosion progression	Estimate (SE); *p* value	0.0002 (0.0001); 0.004	0.287 (0.085); 0.001	–	0.0001 (0.0000); 0.008	0.354 (0.089); < 0.001	–
Total radiographic progression ^b^	Estimate (SE); *p* value	0.0004 (0.0001); < 0.001	0.930 (0.154); < 0.001	1.552 (0.846); 0.072	0.0002 (0.0001); 0.008	1.099 (0.222); < 0.001	− 0.498 (0.786); 0.529
Radiographic erosion progression	Estimate (SE); *p* value	0.0001 (0.0000); 0.011	0.182 (0.092); 0.052	0.531 (0.417); 0.208	0.0001 (0.0000); 0.048	0.220 (0.123); 0.080	− 0.231 (0.382); 0.548
Radiographic JSN progression	Estimate (SE); *p* value	0.0003 (0.0001); < 0.001	0.748 (0.089); < 0.001	1.017 (0.572); 0.080	0.0002 (0.0001); 0.012	0.879 (0.138); < 0.001	− 0.269 (0.533); 0.615

^a^Based on univariate linear regression analysis

^b^Assessed by mTSS

^c^RAMRIQ synovitis is not normalised, where RAMRIQ osteitis and erosions are; as a result, the estimates and standard errors are low due to the scale

*JSN* joint space narrowing, *MRI* magnetic resonance imaging, *mTSS* modified Total Sharp Score, *RA* rheumatoid arthritis, *RAMRIQ* automated quantitative RA MRI assessment system, *RAMRIS* RA MRI scoring system, *SE* standard error

### Early RAMRIQ changes as predictors of RAMRIS erosion progression and radiographic progression

n univariate analyses, lower increases from baseline to both months 1 and 3 in RAMRIQ synovitis and osteitis were significant predictors of smaller changes in RAMRIS erosions from baseline to month 12. Lower changes from baseline to both months 1 and 3 in RAMRIQ synovitis and osteitis (but not RAMRIQ erosions) also significantly predicted less total radiographic progression (change in mTSS) and JSN progression between baseline and month 12 (Table 1).

### Early changes in RAMRIS erosions and RAMRIQ synovitis and osteitis as predictors of radiographic progression

Baseline variables, including age, gender, race and smoking status, were not significant in univariate analyses (data not shown) and therefore were not included in the subsequent multivariate analyses. In multivariate analyses, the change in RAMRIS erosions from baseline to month 1, but not from baseline to month 3, was a significant independent predictor of total radiographic progression from baseline to month 12, and the changes in RAMRIS erosions from baseline to both months 1 and 3 were significant independent predictors of radiographic erosion progression from baseline to month 12 (Table 2). Furthermore, changes in RAMRIQ osteitis (but not RAMRIQ synovitis) at both months 1 and 3 were significant independent predictors of total and JSN radiographic progression from baseline to month 12, and the change in RAMRIQ synovitis at month 1, but not at month 3, was a significant independent predictor of radiographic erosion progression from baseline to month 12 (Table 2).

**Table 2:** Multivariate analyses^a^ of predictive value of early MRI changes for later radiographic changes

		Change from baseline to month 1			Change from baseline to month 3		
Change from baseline to month 12		RAMRIQ synovitis	RAMRIQ osteitis	RAMRIS erosions	RAMRIQ synovitis	RAMRIQ osteitis	RAMRIS erosions
Total radiographic progression^b^	Estimate	0.0002	0.645	0.777	0.0001	0.867	0.434
	*p* value	0.148	0.002	0.043	0.407	0.001	0.073
Radiographic erosion progression	Estimate	0.0001	− 0.043	0.590	0.0001	− 0.001	0.425
	*p* value	0.040	0.705	0.007	0.145	0.996	0.001
Radiographic JSN progression	Estimate	0.0000	0.688	0.187	0.0000	0.868	0.009
	*p* value	0.647	< 0.001	0.409	0.907	< 0.001	0.954

^a^Based on multivariate linear regression analysis

^b^Assessed by mTSS

*JSN* joint space narrowing, *MRI* magnetic resonance imaging, *mTSS* modified Total Sharp Score, *RA* rheumatoid arthritis, *RAMRIQ* automated quantitative RA MRI assessment system*, RAMRIS* RA MRI scoring system

### Early changes in disease activity as predictors of radiographic progression

In univariate analyses, there was no significant predictive value (*p* values > 0.05 for all analyses) of changes from baseline to month 1 or 3 in CDAI or DAS28-4(ESR) with respect to radiographic progression from baseline to month 12 (Additional file 2).

## Discussion

Data from an RA clinical trial with a small number of patients, pooled across treatment groups, demonstrated that MRI assessment at months 1 and 3 after treatment initiation, using RAMRIS or the automated system RAMRIQ, generally predicted radiographic disease progression from baseline to month 12. Of the changes assessed, changes as early as month 1 in RAMRIQ synovitis and osteitis and in RAMRIS erosions were the best predictors of later disease progression. No predictive value was observed for concomitant changes from baseline to months 1 and 3 in the CDAI and DAS28-4(ESR) disease activity measures and later radiographic progression.

A number of studies have looked at baseline MRI features predicting outcome. An observational study of patients with RA of < 1 year’s duration reported that RAMRIS-assessed osteitis at baseline was associated with radiographic progression at 1 year [11], and in a randomised controlled trial in patients with early RA, baseline osteitis assessed with RAMRIS was a strong predictor of radiographic progression after 2 years [12]. In terms of response prediction, in a clinical trial of golimumab in MTX-naïve patients with RA, multivariate logistic regression analysis of RAMRIS-assessed changes showed synovitis and osteitis at baseline and after 24 weeks of treatment predicted radiographic progression at 12 months, as did an increase in erosion of > 0.5 at weeks 12 and 24; regression models incorporating baseline and 12- and 24-week changes in MRI measures of synovitis and osteitis improved the prediction of radiographic progression at 12 months above clinical disease activity measures [13]. However, we believe this to be the first report of MRI-detected changes as early as 1 month predicting radiographic progression after 12 months.

Given that it has been demonstrated that there is a window of opportunity in RA, an early predictor of disease progression at month 12 could allow treatment decisions to be made sooner, in line with the recommended treat-to-target approach in RA [2, 3]. The most effective early RA intervention will ideally lead to reduced long-term impact of the disease; thus, early identification of the likely long-term efficacy of treatment provides potential patient benefit [14]. Early prediction of longer-term structural treatment benefit might also allow clinical trials of shorter duration and with more limited sample size, focused on structural changes, to be designed to assess new agents for the treatment of RA. Such trials would be preferable from the patient perspective and could also reduce drug development costs by rapidly assessing the clinical response to new therapies and reducing the need for additional imaging studies.

Early changes in disease activity, as measured with CDAI and DAS28-4(ESR), showed no significant predictive value for subsequent structural damage progression. Thus, the analyses presented here demonstrated that MRI assessment of structural changes during the first 3 months of treatment for RA, and even as early as 1 month, may better predict patients’ radiographically assessed response to treatment by month 12 than changes in clinical measures of disease activity in those 3 months. The MRI scoring procedures included here can be implemented using widely available MRI equipment (1.5-T scanners) and imaging sequences, as described previously [8].

A number of limitations of this study are acknowledged. The small sample size, post hoc nature of the analysis, single RAMRIS reader and lack of independence of the predictive parameters assessed all indicate a need for caution in the interpretation of findings. These analyses were exploratory in nature with the intent to determine which parameters had the strongest ability to predict treatment response. While this analysis, based on clinical trial data, demonstrated the utility of the RAMRIQ technique, the technology is still under development. Implementation in non-clinical-trial settings may be subject to variability due to potential variability in the implementation of the RAMRIS/RAMRIQ protocol across different magnetic field strengths, and possible inter-operator differences. Further exploration of this finding with other agents is warranted to evaluate the clinical implications.

## Conclusions

In conclusion, incorporation of early MRI measurement in RA clinical trials will likely enable prediction of later response to treatment, allowing more rapid assessment of the efficacy of new medications. There is also the potential to use MRI as a biomarker to inform treatment decisions in clinical practice. However, the findings reported here require further validation.

## Supplementary information

**Additional file 1:** Descriptive statistics of mTSS and statistical analysis of change from baseline in mTSS over time.

**Additional file 2:** Univariate analyses^a^ of predictive value of early changes in CDAI and DAS28-4(ESR) for later radiographic changes.

## Data Availability

Upon request, and subject to certain criteria, conditions and exceptions (see https://www.pfizer.com/science/clinical-trials/trial-data-and-results for more information), Pfizer will provide access to individual de-identified participant data from Pfizer-sponsored global interventional clinical studies conducted for medicines, vaccines and medical devices (1) for indications that have been approved in the US and/or EU or (2) in programmes that have been terminated (i.e., development for all indications has been discontinued). Pfizer will also consider requests for the protocol, data dictionary, and statistical analysis plan. Data may be requested from Pfizer trials 24 months after study completion. The de-identified participant data will be made available to researchers whose proposals meet the research criteria and other conditions, and for which an exception does not apply, via a secure portal. To gain access, data requestors must enter into a data access agreement with Pfizer.

## Abbreviations

BID: Twice daily
CDAI: Clinical Disease Activity Index
DAS28-4(ESR): Disease Activity Score in 28 joints, erythrocyte sedimentation rate
DMARD: Disease-modifying antirheumatic drug
JAK: Janus kinase
JSN: Joint space narrowing
MCP: Metacarpophalangeal
MRI: Magnetic resonance imaging
mTSS: Modified Total Sharp Scores
MTX: Methotrexate
OMERACT: Outcome Measures in Rheumatology
RAMRIQ: Automated quantitative RA MRI assessment system
RAMRIS: RA MRI scoring system
SE: Standard error

## References

[1] van der Heijde D, Tanaka Y, Fleischmann R, Keystone E, Kremer J, Zerbini C (2013). Tofacitinib (CP-690,550) in patients with rheumatoid arthritis receiving methotrexate: twelve-month data from a twenty-four-month phase III randomized radiographic study. Arthritis Rheum.

[2] Singh JA, Saag KG, Bridges SL, Akl EA, Bannuru RR, Sullivan MC (2016). 2015 American College of Rheumatology guideline for the treatment of rheumatoid arthritis. Arthritis Rheumatol.

[3] Smolen JS, Landewé R, Breedveld FC, Dougados M, Emery P, Gaujoux-Viala C (2010). EULAR recommendations for the management of rheumatoid arthritis with synthetic and biological disease-modifying antirheumatic drugs. Ann Rheum Dis.

[4] White D, Pahau H, Duggan E, Paul S, Thomas R (2013). Trajectory of intensive treat-to-target disease modifying drug regimen in an observational study of an early rheumatoid arthritis cohort. BMJ Open.

[5] Skacelova S, Pavelka K, Chroust K (2013). Early response to infliximab therapy predicts long-term disease activity outcomes in patients with rheumatoid arthritis: results from the Czech National Biological Therapy Registry (ATTRA). Ann Rheum Dis.

[6] Kremer J, Dougados M, Genovese MC, Emery P, Yang L, de Bono S (2015). Response to baricitinib at 4 weeks predicts response at 12 and 24 weeks in patients with rheumatoid arthritis: results from two phase 3 studies. Arthritis Rheumatol.

[7] Østergaard M, Peterfy C, Conaghan P, McQueen F, Bird P, Ejbjerg B, et al. OMERACT Rheumatoid Arthritis Magnetic Resonance Imaging Studies. Core set of MRI acquisitions, joint pathology definitions, and the OMERACT RA-MRI scoring system. J Rheumatol. 2003;30:1385–6. 12784422

[8] Conaghan PG, Østergaard M, Bowes MA, Wu C, Fuerst T, van der Heijde D (2016). Comparing the effects of tofacitinib, methotrexate and the combination, on bone marrow oedema, synovitis and bone erosion in methotrexate-naive, early active rheumatoid arthritis: results of an exploratory randomised MRI study incorporating semiquantitative and quantitative techniques. Ann Rheum Dis.

[9] Bowes MA, Guillard G, Gill E, Vincent GR, Hensor E, Freeston JE (2014). Novel quantification of MRI provides a more sensitive outcome measure than Ramris. Arthritis Rheumatol.

[10] van der Heijde D, Simon L, Smolen J, Strand V, Sharp J, Boers M (2002). How to report radiographic data in randomized clinical trials in rheumatoid arthritis: guidelines from a roundtable discussion. Arthritis Rheum.

[11] Haavardsholm EA, Bøyesen P, Østergaard M, Schildvold A, Kvien TK (2008). Magnetic resonance imaging findings in 84 patients with early rheumatoid arthritis: bone marrow oedema predicts erosive progression. Ann Rheum Dis.

[12] Hetland ML, Ejbjerg B, Hørslev-Petersen K, Jacobsen S, Vestergaard A, Jurik AG (2009). MRI bone oedema is the strongest predictor of subsequent radiographic progression in early rheumatoid arthritis. Results from a 2-year randomised controlled trial (CIMESTRA). Ann Rheum Dis.

[13] Baker JF, Ostergaard M, Emery P, Hsia EC, Lu J, Baker DG (2014). Early MRI measures independently predict 1-year and 2-year radiographic progression in rheumatoid arthritis: secondary analysis from a large clinical trial. Ann Rheum Dis.

[14] Raza K, Filer A (2015). The therapeutic window of opportunity in rheumatoid arthritis: does it ever close?. Ann Rheum Dis.

