# Supplementary material for: Very early MRI responses to therapy as a predictor of later radiographic progression in early rheumatoid arthritis
Source: Arthritis Res Ther. 2019 Oct 21;21:214. doi: 10.1186/s13075-019-2000-1 (PMC6805378; doi:10.1186/s13075-019-2000-1)
Supplement: Supplementary file 1 — Additional file 1: Descriptive statistics of mTSS and statistical analysis of change from baseline in mTSS over time. [file 13075_2019_2000_MOESM1_ESM.docx]

**Additional file 1:** Descriptive statistics of mTSS and statistical analysis of change from baseline in mTSS over time

|  | Tofacitinib 10 mg BID + MTX | Tofacitinib 10 mg BID monotherapy | MTX  monotherapy |
| --- | --- | --- | --- |
| mTSS, mean (SD) |  |  |  |
| Baseline | 13.0 (21.7)  *n* = 34 | 12.6 (26.0)  *n* = 36 | 13.7 (26.0)  *n* = 37 |
| Month 6 | 13.8 (23.1)  *n* = 29 | 13.0 (28.6)  *n* = 27 | 8.3 (15.6)  *n* = 28 |
| Month 12 | 14.6 (23.1)  *n* = 26 | 8.6 (11.0)  *n* = 25 | 9.7 (17.6)  *n* = 22 |
| Change from baseline mTSS,  least squares mean (SE)^a^ |  |  |  |
| Month 6 | 0.44 (0.50)  *n* = 29 | -0.14 (0.51)  *n* = 27 | 0.93 (0.52)  *n* = 28 |
| Month 12 | 0.85 (0.51)  *n* = 26 | -0.15 (0.52)  *n* = 25 | 1.36 (0.54)  *n* = 22 |

^a^Data are from a mixed effect model for repeated measures, incorporating all time points, and including treatment arms as factor and baseline as a covariate.

*BID* twice daily, *mTSS* modified Total Sharp Score, *MTX* methotrexate, *n* number of evaluable patients, *SD* standard deviation, *SE* standard error.
