# Supplementary material for: Very early MRI responses to therapy as a predictor of later radiographic progression in early rheumatoid arthritis
Source: Arthritis Res Ther. 2019 Oct 21;21:214. doi: 10.1186/s13075-019-2000-1 (PMC6805378; doi:10.1186/s13075-019-2000-1)
Supplement: Supplementary file 2 — Additional file 2: Univariate analysesa of predictive value of early changes in CDAI and DAS28-4(ESR) for later radiographic changes. [file 13075_2019_2000_MOESM2_ESM.docx]

**Additional file 2:** Univariate analyses^a^ of predictive value of early changes in CDAI and DAS28-4(ESR) for later radiographic changes

|  |  | Change from baseline to Month 1 | | Change from baseline to Month 3 | |
| --- | --- | --- | --- | --- | --- |
| Change from baseline to Month 12 |  | CDAI | DAS28-4(ESR) | CDAI | DAS28-4(ESR) |
| Total  radiographic  progression^b^ | Estimate  (SE)  *p* value | 0.081  (0.041)  0.051 | 0.616  (0.424)  0.150 | 0.044  (0.033)  0.195 | 0.317  (0.339)  0.352 |

^a^Based on univariate linear regression analysis.

^b^Assessed by mTSS.
*CDAI* clinical disease activity index; *DAS28-4(ESR)* disease activity score in 28 joints, erythrocyte sedimentation rate; *MRI* magnetic resonance imaging, *mTSS* modified Total Sharp Score, *RA* rheumatoid arthritis, *SE* standard error.
